# Comment on “First finding of continental deep subduction in the Sesia Zone of Western Alps and implications for subduction dynamics”

**DOI:** 10.1093/nsr/nwae454

**Published:** 2024-12-12

**Authors:** Roberto Compagnoni, Mattia Gilio, Stefano Ghignone, Emanuele Scaramuzzo, Alessia Borghini, Marco Bruno

**Affiliations:** Department of Earth Sciences, University of Turin, Italy; Department of Earth and Environmental Sciences, University of Pavia, Italy; Department of Earth Sciences, University of Turin, Italy; Department of Science and High Technology, University of Insubria, Italy; Faculty of Geology, Geophysics and Environmental Protection, AGH University of Krakow, Poland; Department of Earth Sciences, University of Turin, Italy

In their paper, Chen *et al.* [1] claim the finding of a new coesite-bearing locality on the Western Alps, in the Sesia Zone. Coesite was allegedly found in garnet-bearing orthogneiss from the Eclogitic Micaschist Complex (EMC; see e.g. [2,3] and references therein). They described coesite as a partly preserved polymineralic inclusion stored in garnet, made by albite, quartz and few µm-sized coesite relicts or only by quartz and few µm-sized coesite. The authors characterized the inclusions via µ-Raman spectroscopy and asserted the occurrence of coesite based solely on the presence of a single peak at 521 cm^−1^ (Figure 4 in Chen *et al.* [1]). The authors provide new *P-T* estimates for the ultra-high pressure (UHP) metamorphic peak using thermodynamic modelling: *P* = 2.8–3.3 GPa and T = 450–520°C, according to their supposed coesite finding.

In this comment, we question the coesite occurrence, as we believe that it is not supported by the microstructural evidence and by the Raman spectra provided. Coesite is the most important UHP index mineral and its presence in metamorphic rocks indicates subduction at a depth of >100 km. Hence, it has important implications on the reconstruction of the tectonometamorphic evolution of subduction-accretionary systems (see e.g. [4,5]). Therefore, new coesite findings should be properly documented.

Raman spectrometry is widely used for identifying both organic and inorganic compounds, especially in cases where the unknown phase is small or involves polymorphs. Rock-forming minerals are often identified by comparing the obtained spectrum with reliable mineral databases. Given that most silicate minerals have vibrational modes between 15 and 1300 cm⁻¹, peak overlaps between different phases are unavoidable. Therefore, a mineral phase should be identified from a set of representative peaks rather than a single peak. The reported spectra should cover at least the 100–1300 cm⁻¹ range.

Chen *et al.* [1] claim to have identified coesite in garnet within two quartz + albite inclusions and at the rim of a quartz inclusion. However, Fig. 4A and 4C show that the analysed spots are at the contact between the exposed quartz inclusions and the host garnet, areas often contaminated by dirt or polishing material, which should be avoided for mineral identification.

The main problem in the Raman mineral identification performed by Chen *et al.* [1] is that the spectra are poorly characterized: some peaks are left unidentified while others are incorrectly assigned, as we will try to highlight below. The A_1_ mode of quartz at 465 cm⁻¹ is visible in all spectra, alongside the E mode at 128 cm⁻¹ and the A_1_ mode at 207 cm⁻¹. However, the authors also assign a strong peak at 354 cm⁻¹ to quartz. While quartz has an A_1_ mode near this wavenumber, its intensity should be much lower (about a tenth of the 465 cm⁻¹ peak [6]). Therefore, this is likely a misinterpretation. The authors correctly identify albite peaks near 290 and 506 cm⁻¹ but overlook the peak at 478 cm⁻¹ and two weak peaks between 150 and 200 cm⁻¹, which also belong to albite [7]. The authors interpret the peak at 521 cm⁻¹ as the A_g_ mode of coesite. However, other characteristic coesite peaks, particularly the one at 271 cm⁻¹, are missing. This peak should have about one-third the intensity of the main 521 cm⁻¹ peak [8]. Additionally, the three coesite peaks between 100 and 210 cm⁻¹, which should be recognizable, are absent. The peak at 327 cm⁻¹, attributed to coesite, is also misinterpreted. If this peak had measurable intensity, the 521 cm⁻¹ peak should be much stronger. Garnet has up to five peaks between 300 and 400 cm⁻¹, whose positions vary slightly with composition [9]. For instance, andradite has peaks at 321 and 325 cm⁻¹, with other modes at 352 and 370 cm⁻¹, all of which match the spectra in Fig. 4B–D. Garnet also has an A_1__g_ mode between 515 and 570 cm⁻¹, which could correspond to the 521 cm⁻¹ peak [10]. The missing 700–1300 cm⁻¹ spectral window makes identifying the garnet composition difficult, but the 521 cm⁻¹ peak likely belongs to garnet rather than coesite.

In summary, the spectra reported by Chen *et al.* [1] are poorly characterized, leading to misidentifications of coesite in the mixed quartz + albite + garnet Raman spectra.

Based on the above discussion, it is evident that the new data presented in the article by Chen *et al.* [1] are not sufficient to confidently report the presence of coesite in the EMC. The occurrence of coesite is in fact based only on the interpretation of Raman spectra (see e.g. [10] and references therein). However, we believe that the proposed Raman spectra were improperly interpreted. The unusual occurrence of coesite at the rim of the inclusions instead of in the centre surrounded by quartz as commonly observed (see e.g. [10] and references therein) and the absence of the textural features typical of coesite inversion to quartz makes the evidence of the presence of coesite even weaker.

Additionally, the pseudosection proposed by Chen *et al.* [1] is incorrect, because it is based on the wrong assumption of considering coesite stable with the peak-P stable mineral assemblage. Consequently, their PT reconstruction is also incorrect, as the peak-P lies below UHP metamorphic conditions.

Based on all the arguments listed above, we believe that the finding of coesite in EMC is not convincing as it is. Hence, the jadeite-bearing orthogneiss from EMC of the lower Aosta Valley experienced most likely the entire *P-T-t* evolution within the quartz stability field as further confirmed by the recent paper by Gilotti *et al.* [3].
